# Early antithrombotic therapy improves 90-day functional outcomes in patients with postinterventional cerebral hyperdensity after mechanical thrombectomy: a retrospective cohort study

**DOI:** 10.3389/fneur.2026.1891162

**Published:** 2026-10-23

**Authors:** Yunhe Luo, Jiang Zhao, Ligang Huang, Shiliang Xia, Shidong Wei, Ting Ni, Wenjie Cao, Chunying Shen, Jin Zheng

**Affiliations:** 1 Department of Neurology, Minhang Hospital, Fudan University, Shanghai, China; 2 Department of Neurology, Huashan Hospital, Fudan University, Shanghai, China; 3 Shanghai Sixth People's Hospital, Shanghai Jiao Tong University, Shanghai, China; 4 Human Phenome Institute, Fudan University, Shanghai, China; 5 Department of Pharmacy, Minhang Hospital, Fudan University, Shanghai, China

**Keywords:** 90-day functional outcome, acute ischemic stroke, antithrombotic therapy, mechanical thrombectomy, postinterventional cerebral hyperdensity

## Abstract

**Background and purpose:**

Postinterventional cerebral hyperdensity (PCHD) is a specific type of hyperdensity commonly observed on computed tomography (CT) scans of the head after mechanical thrombectomy (MT). This study aimed to investigate the association between early antithrombotic therapy (EAT) and 90-day functional outcomes in anterior circulation acute ischemic stroke (acAIS) patients with PCHD.

**Methods:**

A retrospective cohort study was conducted on 141 consecutive acAIS patients with PCHD after MT. EAT was defined as antithrombotic therapy administered within 24 h post-MT. The primary outcome was a favorable 90-day functional outcome (modified Rankin Scale (mRS) score of 0–2). The secondary outcomes included 90-day mortality, ΔmRS (defined as the mRS score before MT minus the 90-day mRS score), enlargement of cerebral hyperdensity, and persistent hyperdensity on the 24-h follow-up CT graded by the European Cooperative Acute Stroke Study (ECASS III) morphology. Baseline and outcome data were compared between the EAT and non-EAT groups. Univariate and multivariate logistic regression analyses were performed to assess the association between EAT and favorable outcomes. Sensitivity and subgroup analyses were performed to evaluate the robustness and internal consistency of the results.

**Results:**

Of the 129 patients with complete 90-day mRS data, 59 (45.7%) received EAT. The EAT group had a higher proportion of favorable outcomes (59.3% vs. 37.1%, *p* = 0.019). A multivariate logistic regression analysis showed that EAT remained significantly associated with favorable outcomes (Model 1: odds ratio (OR) 2.469, 95% confidence interval (CI) 1.195–5.104, *p* = 0.015; Model 2: OR 2.72, 95% CI 1.02–7.20, *p* = 0.045; Model 3: OR 3.68, 95% CI 1.32–10.30, *p* = 0.013), and the results were consistent after imputation based on discharge data and multiple imputation. Sensitivity and subgroup analyses showed consistent results, with no significant interaction effects observed.

**Conclusion:**

EAT is associated with improved 90-day functional outcomes in acAIS patients with PCHD, warranting further investigation in larger prospective studies.

## Introduction

Mechanical thrombectomy (MT) is currently the most effective treatment for acute ischemic stroke (AIS) caused by large artery occlusion (1). However, hyperdensity is frequently observed on computed tomography (CT) scans of the head following MT, with an estimated incidence of up to 86% in AIS patients (2). This hyperdensity may reflect contrast extravasation or hemorrhagic transformation (HT). Notably, research indicates that approximately 20% of patients experience neurological deterioration (ND) within 72 h post-MT (3). ND is an independent risk factor for poor functional outcomes at 3 months (4, 5). Early administration of antithrombotic drugs may prevent reocclusion and mitigate microcirculatory disturbances, a phenomenon often referred to as the “no-reflow phenomenon” (6–8). Nevertheless, the early use of antithrombotic medications remains limited due to concerns about hyperdensity (1).

Postinterventional cerebral hyperdensity (PCHD) has recently been proposed as a term referring to cerebral parenchymal hyperdensity without a space-occupying effect, excluding relatively severe HT (9). It differs from a similar concept, post-MT intraparenchymal hyperdensity (PTIH) (10), as PTIH does not exclude parenchymal hyperdensity with a space-occupying effect.

Considering that the pathological basis of HT is distinct from that of intracerebral hemorrhage (11, 12), which is primarily due to the rupture of small aneurysms (13), PCHD may be associated with a lower risk of increasing bleeding. Moreover, some studies have shown the safety of antithrombotic therapy in patients with asymptomatic hemorrhagic transformation (14, 15). Additionally, the 2018 Guidelines for the Early Management of AIS Patients stated that the decision to initiate or continue antiplatelet or anticoagulant therapy can be made based on clinical evaluation (IIB/-NR) (1). In a similar vein, some postoperative management teams in our center have been proactively administering early antithrombotic therapy (EAT) within the first 24 h postoperatively to patients with PCHD in recent years.

Our previous study revealed that EAT may reduce the incidence of ND in anterior circulation thrombectomy patients with PCHD (16). However, PCHD remains underappreciated and may serve as a valuable criterion for identifying patients with post-MT hyperdensity who could benefit from EAT. Therefore, we conducted this retrospective study to explore whether EAT is associated with improved 90-day functional outcomes in anterior circulation thrombectomy patients with PCHD.

## Patients and methods

### Patient recruitment

We performed a retrospective analysis of all AIS patients who underwent MT at our hospital, a tertiary teaching hospital affiliated with Fudan University, from January 2019 to April 2023 (before the establishment of the combined postoperative management teams). The inclusion criteria were as follows: (1) patients who were aged 18 years or older; (2) patients with anterior circulation infarction; (3) patients with premorbid modified Rankin Scale (mRS) scores of ≤2; and (4) patients with the presence of hyperdensity on a brain CT scan obtained immediately after MT, consistent with the criteria for PCHD (9), as defined in the Imaging Analysis section. Patients were excluded if their data were unavailable. This study was reviewed and approved by the Ethical Review Board of our hospital.

### Data collection and imaging analysis

Demographic, clinical, and procedural data were extracted primarily from the database of the Minhang Stroke Registry. Demographic data included age, sex, comorbidities (hypertension, diabetes mellitus, dyslipidemia, atrial fibrillation), and smoking history. Clinical data included the National Institutes of Health Stroke Scale (NIHSS) score before MT, the NIHSS score after MT, stroke etiology according to the Trial of ORG 10172 in Acute Stroke Treatment (TOAST) classification, intravenous thrombolysis (IVT) before MT, initial occlusion site, MT technique, TICI scores, and stent implantation. Procedural data included door-to-puncture time (DPT), onset-to-final TICI score time (OFT), and the number of retrieval attempts.

PCHD was defined as non-solid, visually distinct parenchymal hyperdense areas identified on the first postinterventional CT immediately following MT, with a surface area of at least 0.1 cm^3^, an increased density of at least 5 Hounsfield units (HU) compared to the unaffected contralateral side, without a space-occupying effect, and conforming to the gray-white matter boundaries of typical anatomical structures (9).

Lesions with a space-occupying effect (PH1/PH2) or located outside the brain parenchyma, as well as any intracranial hemorrhage present on the pre-procedural CT, were excluded at enrollment (*n* = 57). On the immediate post-MT CT, PCHD was classified according to the adapted European Cooperative Acute Stroke Study (ECASS III) criteria as hemorrhagic infarction HI1 or HI2. Because contrast extravasation and hemorrhage cannot be reliably distinguished without a dual-energy acquisition, the 24-h follow-up CT was additionally classified according to ECASS III morphology (HI1, HI2, PH1, or PH2) to characterize persistent hyperdensity.

EAT was defined as the administration of antithrombotic drugs within 24 h postoperatively, which included antiplatelet therapy and anticoagulant therapy. Before May 2023, following the merger of our postoperative management teams, EAT strategies for PCHD patients were applied to some patients within 24 h, whereas others received antithrombotic medications after the 24-h follow-up CT or even after later CT re-examination, because some attendings in our department were also worried about the safety and efficacy of EAT in AIS patients with PCHD. Antithrombotic therapy in patients with PCHD following mechanical thrombectomy (MT) was administered according to the following protocol (16). In patients with PCHD following mechanical thrombectomy (MT), antithrombotic therapy was administered according to the following protocol. Considering the hyperdensity without a space-occupying effect on the computed tomography (CT) and the intraoperative heparinization, tirofiban (a maintenance dose of 0.2–0.3 mg/h for approximately 24 h without a loading dose, with a 4–6 h overlap with oral dual antiplatelet therapy) was given only to patients with stent implantation and residual severe stenosis. For other atherosclerotic occlusion patients, oral dual antiplatelet therapy without a loading dose was given as soon as possible within 24 h. For cardioembolic patients, oral antiplatelet therapy without a loading dose was administered. Compared with dual antiplatelet therapy, single antiplatelet therapy was applied only to some cardioembolic patients with severe stroke symptoms and a low-density area on CT involving > 1/3 of the vascular territory after MT. The timing of oral anticoagulant therapy initiation (with low-molecular-weight heparin overlap in some cases, especially when warfarin was used) was determined by residual infarction volume and the stroke severity after MT. Oral antiplatelet agents were chosen from aspirin (100 mg/day), clopidogrel (75 mg/day), and cilostazol (100 mg twice daily, with a starting dose of 50 mg twice daily). Aspirin combined with clopidogrel was used as the conventional oral dual antiplatelet therapy. When aspirin or clopidogrel was contraindicated, cilostazol was prescribed as an alternative. When single oral antiplatelet therapy was chosen, the sequence of drug applications was aspirin, clopidogrel, and cilostazol. Oral anticoagulant agents used in our department included warfarin (with low-molecular-weight heparin overlap for at least 5 days and dose adjustment to achieve an international normalized ratio [INR] of 2–3), dabigatran (110 mg twice daily), and rivaroxaban (15 mg/day). Warfarin was usually used in patients with valvular atrial fibrillation, while either dabigatran or rivaroxaban was used in patients with non-valvular atrial fibrillation. Low-molecular-weight heparin used in our department included enoxaparin (20–40 mg twice daily) and nadroparin (2050–4,100 anti-Xa units twice daily). In particular, it is worth noting that these antithrombotic strategies for PCHD patients were applied to some patients within 24 h, whereas others received antithrombotic medications after a 24-h follow-up CT or even after later CT re-examination, reflecting concerns among some attending physicians in our department regarding the safety and efficacy of early antithrombotic therapy in acute ischemic stroke patients with PCHD.

A cranial non-contrast CT scan was routinely performed upon admission, immediately after MT, and during postoperative follow-up. Low density on immediate cranial non-contrast CT after MT was used to assess postinterventional ischemia. Massive cerebral infarction was defined as a low-density area on immediate post-MT CT exceeding one-third of the vascular territory. Early brain edema was defined as visible swelling on CT within 72 h postoperatively, characterized by hypodense areas with loss of gray-white matter differentiation, effacement of sulci, and compression of ventricles. All images were analyzed by two experienced neurologists (YL and LH) who were blinded to clinical characteristics and study outcomes. In cases of disagreement, images were reviewed until a consensus was reached.

### Clinical outcomes

The primary outcome was a favorable 90-day functional outcome, defined as an mRS score of 0–2. The secondary outcomes included 90-day mortality, ΔmRS (defined as the mRS score before MT minus the 90-day mRS), enlargement of cerebral hyperdensity, and persistent hyperdensity on the 24-h follow-up CT. Mortality was defined as an mRS score of 6. Enlargement of cerebral hyperdensity was defined as a visible increase in hyperdensity volume on follow-up non-contrast CT during hospitalization.

### Statistical analysis

Statistical analyses were performed using R (version 4.4.1, R Foundation for Statistical Computing, Vienna, Austria). The primary analysis included all patients with complete data on demographics, clinical characteristics, imaging findings, and 90-day functional outcomes. Patients were divided into two groups based on whether they received EAT: the EAT group and the non-EAT group. Additionally, patients were categorized according to their 90-day functional outcomes into a favorable outcome group (mRS score 0–2) and an unfavorable outcome group (mRS score >2).

### Primary analysis

Continuous data were reported as mean ± standard deviation (SD) for normally distributed variables and as median (interquartile range, IQR) for non-normally distributed variables. Comparisons between the groups were performed using the t-test for normally distributed continuous data and the Mann–Whitney U-test for non-normally distributed continuous data. Categorical variables were presented as counts (percentages) and compared using Fisher’s exact test or Pearson’s χ^2^ test, as appropriate.

Three nested logistic regression models were constructed to assess the association between EAT and 90-day functional outcomes. Model 1 adjusted for age and sex. Model 2 additionally adjusted for low density on the immediate post-MT CT, early brain edema, IVT before MT, dyslipidemia, the NIHSS score before MT, the number of retrieval attempts, and post-procedural PCHD type (HI2 vs. HI1). Model 3 replaced the NIHSS score before MT with the NIHSS score after MT. Adjusted covariates were specified *a priori* from established prognostic factors and clinical determinants of EAT use; univariate results with a *p*-value of less than 0.10 were reviewed as a reference but were not used as the sole selection criterion. A multivariate logistic regression analysis was performed for all models to evaluate the robustness of the association across different levels of adjustment. Apart from early brain edema, assessed within 72 h of MT, all covariates were measured before EAT administration, and no variables measured at 24 h or later were entered as covariates. Sensitivity analyses repeated the models after omitting early brain edema and after restricting the analysis to baseline variables measured before treatment.

### Sensitivity analysis

To assess the robustness of our findings, two imputation strategies were used for the 12 patients with missing 90-day mRS data. First, the discharge mRS score was carried forward as a single imputation. Second, multiple imputation by chained equations (MICE) was performed with 20 imputed datasets, and estimates were combined by Rubin’s rules. Primary analyses were repeated using the imputed datasets to evaluate the consistency of the results. The 24-h CT outcome does not depend on the 90-day mRS scores and was analyzed in all 135 patients with an interpretable 24-h CT; the 6 patients without a 24-h CT were additionally included in the multiple imputation model.

### Subgroup analysis

Subgroup analyses were performed to explore the consistency of the association between EAT and 90-day functional outcomes across different patient characteristics, including old age (≥80 years), sex, atrial fibrillation, IVT before MT, residual stenosis, and large artery atherosclerosis (LAA) as the cause of stroke.

Since the secondary outcomes and subgroup analyses were exploratory and intended for reference rather than to draw definitive conclusions, no adjustment for multiple comparisons was performed. A *p*-value of <0.05 (two-sided) was considered statistically significant. Convergence and the distribution of the multiple imputation chains were examined as a diagnostic check (Supplementary Figure S1).

## Results

### Patient enrollment and baseline characteristics

The retrospective cohort study ultimately included 141 patients with anterior circulation stroke who underwent MT and presented with PCHD, as shown in Figure 1. As detailed in Table 1, of the 129 patients with available 90-day mRS data, 59 (45.7%) received EAT. Twelve patients lacked 90-day mRS follow-up data; their discharge mRS scores were carried forward in a discharge-imputation analysis (*n* = 141), and MICE (20 imputed datasets, combined using Rubin’s rules) was used as a sensitivity analysis. A total of 135 patients underwent 24-h follow-up CT scans (whereas 6 did not undergo 24-h CT). The NIHSS score after MT was obtained from 117 patients and was used in Model 3.

**Figure 1:**
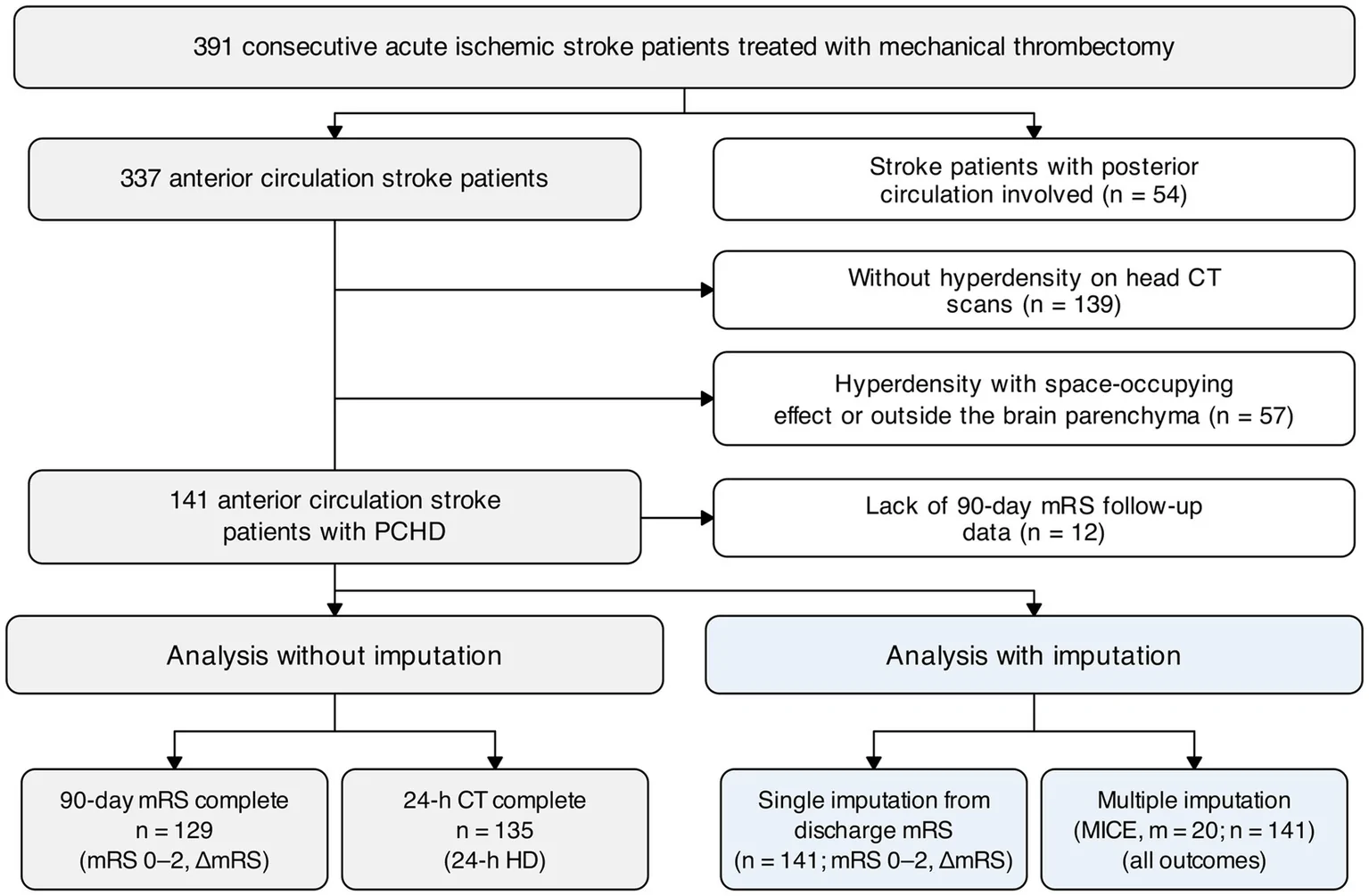
Flowchart of study patient selection. CT, computed tomography; HD, hyperdensity; ΔmRS, change in modified Rankin Scale score (mRS before mechanical thrombectomy minus 90-day mRS); MICE, multiple imputation by chained equations; mRS, modified Rankin Scale; PCHD, postinterventional cerebral hyperdensity.

**Table 1:** Baseline characteristics and outcomes comparison between the early antithrombotic therapy (EAT) and non-EAT groups.

Characteristic	Analysis before imputing mRS scores				Analysis after imputing with discharge mRS scores			
	Total (*n* = 129)	non-EAT (*n* = 70)	EAT (*n* = 59)	*p*-value	Total (*n* = 141)	non-EAT (*n* = 77)	EAT (*n* = 64)	*p*-value
Demographics								
Female, *n* (%)	49 (38.0)	28 (40.0)	21 (35.6)	0.740	53 (37.6)	31 (40.3)	22 (34.4)	0.587
Age, (median [IQR])	72.00 [66.00, 83.00]	72.00 [67.00, 85.75]	73.00 [60.00, 79.50]	0.069	72.00 [66.00, 82.00]	72.00 [67.00, 84.00]	73.50 [60.00, 79.25]	0.099
Hypertension, *n* (%)	80 (62.0)	43 (61.4)	37 (62.7)	1.000	85 (60.3)	45 (58.4)	40 (62.5)	0.751
Diabetes mellitus, *n* (%)	39 (30.2)	23 (32.9)	16 (27.1)	0.607	43 (30.5)	24 (31.2)	19 (29.7)	0.995
Dyslipidemia, *n* (%)	39 (30.2)	22 (31.4)	17 (28.8)	0.897	43 (30.5)	24 (31.2)	19 (29.7)	0.995
Atrial fibrillation, *n* (%)	65 (50.4)	38 (54.3)	27 (45.8)	0.431	69 (48.9)	41 (53.2)	28 (43.8)	0.340
Smoking history, *n* (%)				0.384				0.500
Non-smoker	98 (76.0)	56 (80.0)	42 (71.2)		107 (75.9)	61 (79.2)	46 (71.9)	
Former smoker	9 (7.0)	5 (7.1)	4 (6.8)		9 (6.4)	5 (6.5)	4 (6.2)	
Current smoker	22 (17.1)	9 (12.9)	13 (22.0)		25 (17.7)	11 (14.3)	14 (21.9)	
Premorbid mRS, *n* (%)				0.625				0.999
0	81 (62.8)	42 (60.0)	39 (66.1)		90 (63.8)	49 (63.6)	41 (64.1)	
1	31 (24.0)	17 (24.3)	14 (23.7)		31 (22.0)	17 (22.1)	14 (21.9)	
2	17 (13.2)	11 (15.7)	6 (10.2)		20 (14.2)	11 (14.3)	9 (14.1)	
Clinical characteristics								
NIHSS score before MT (median [IQR])	16.00 [12.00, 20.00]	18.00 [14.00, 21.00]	15.00 [11.50, 18.00]	0.006**	16.00 [12.00, 20.00]	17.00 [14.00, 21.00]	15.00 [12.00, 19.25]	0.045*
NIHSS score after MT (median [IQR])	12.00 [7.00, 16.00]	12.00 [10.00, 16.25]	11.00 [6.00, 16.00]	0.275	12.00 [7.50, 17.00]	12.00 [10.00, 16.50]	11.50 [6.00, 17.00]	0.416
mRS scores before MT (median [IQR])	5.00 [4.00, 5.00]	5.00 [4.00, 5.00]	4.00 [4.00, 5.00]	0.070	5.00 [4.00, 5.00]	5.00 [4.00, 5.00]	5.00 [4.00, 5.00]	0.206
PCHD type (ECASS III), *n* (%)				0.029*				0.017*
HI1	35 (27.1)	13 (18.6)	22 (37.3)		36 (25.5)	13 (16.9)	23 (35.9)	
HI2	94 (72.9)	57 (81.4)	37 (62.7)		105 (74.5)	64 (83.1)	41 (64.1)	
IVT before MT, *n* (%)	41 (31.8)	24 (34.3)	17 (28.8)	0.635	46 (32.6)	27 (35.1)	19 (29.7)	0.619
Residual stenosis, *n* (%)	65 (50.4)	31 (44.3)	34 (57.6)	0.183	74 (52.5)	35 (45.5)	39 (60.9)	0.096
Stent implantation, *n* (%)	6 (4.7)	3 (4.3)	3 (5.1)	1.000	6 (4.3)	3 (3.9)	3 (4.7)	1.000
Low density on CT after MT, *n* (%)				0.723				0.769
Without low density on CT	36 (27.9)	19 (27.1)	17 (28.8)		38 (27.0)	20 (26.0)	18 (28.1)	
Low density <1/3 vascular territory	70 (54.3)	40 (57.1)	30 (50.8)		75 (53.2)	43 (55.8)	32 (50.0)	
Low density >1/3 vascular territory	23 (17.8)	11 (15.7)	12 (20.3)		28 (19.9)	14 (18.2)	14 (21.9)	
Initial occlusion site, *n* (%)				0.573				0.694
ICA	42 (32.6)	21 (30.0)	21 (35.6)		51 (36.2)	27 (35.1)	24 (37.5)	
M1	67 (51.9)	40 (57.1)	27 (45.8)		70 (49.6)	41 (53.2)	29 (45.3)	
M2	10 (7.8)	5 (7.1)	5 (8.5)		10 (7.1)	5 (6.5)	5 (7.8)	
Tandem	10 (7.8)	4 (5.7)	6 (10.2)		10 (7.1)	4 (5.2)	6 (9.4)	
MT techniques, *n* (%)				0.789				0.815
SR	5 (3.9)	3 (4.3)	2 (3.4)		6 (4.3)	3 (3.9)	3 (4.7)	
CA	6 (4.7)	4 (5.7)	2 (3.4)		6 (4.3)	4 (5.2)	2 (3.1)	
Combined SR and CA	118 (91.5)	63 (90.0)	55 (93.2)		129 (91.5)	70 (90.9)	59 (92.2)	
TICI, *n* (%)				0.417				0.351
0	2 (1.6)	1 (1.4)	1 (1.7)		2 (1.4)	1 (1.3)	1 (1.6)	
1	5 (3.9)	3 (4.3)	2 (3.4)		5 (3.5)	3 (3.9)	2 (3.1)	
2a	8 (6.2)	2 (2.9)	6 (10.2)		10 (7.1)	3 (3.9)	7 (10.9)	
2b	35 (27.1)	22 (31.4)	13 (22.0)		40 (28.4)	26 (33.8)	14 (21.9)	
3	79 (61.2)	42 (60.0)	37 (62.7)		84 (59.6)	44 (57.1)	40 (62.5)	
Stroke subtype, *n* (%)				0.598				0.424
LAA	51 (39.5)	26 (37.1)	25 (42.4)		59 (41.8)	29 (37.7)	30 (46.9)	
CE	56 (43.4)	34 (48.6)	22 (37.3)		59 (41.8)	37 (48.1)	22 (34.4)	
SOE	7 (5.4)	3 (4.3)	4 (6.8)		7 (5.0)	3 (3.9)	4 (6.2)	
SUE	15 (11.6)	7 (10.0)	8 (13.6)		16 (11.3)	8 (10.4)	8 (12.5)	
Early brain edema, *n* (%)	31 (24.0)	20 (28.6)	11 (18.6)	0.268	36 (25.5)	24 (31.2)	12 (18.8)	0.136
Procedure								
DPT, min, (median [IQR])	90.00 [79.00, 121.00]	89.50 [76.25, 115.00]	92.00 [79.50, 129.50]	0.437	92.00 [79.00, 121.00]	90.00 [76.00, 117.00]	93.00 [79.75, 129.25]	0.568
OFT, min, (median [IQR])	295.00 [202.00, 428.00]	290.00 [205.25, 364.50]	331.00 [211.00, 524.50]	0.203	294.00 [202.00, 413.00]	290.00 [202.00, 366.00]	314.50 [216.50, 499.00]	0.280
Number of retrieval attempts, *n* (%)				0.483				0.527
1	61 (47.3)	33 (47.1)	28 (47.5)		63 (44.7)	34 (44.2)	29 (45.3)	
2	24 (18.6)	15 (21.4)	9 (15.3)		28 (19.9)	18 (23.4)	10 (15.6)	
3	24 (18.6)	10 (14.3)	14 (23.7)		27 (19.1)	12 (15.6)	15 (23.4)	
≥4	20 (15.5)	12 (17.1)	8 (13.6)		23 (16.3)	13 (16.9)	10 (15.6)	
mRS at discharge (median[IQR])	4.00 [3.00, 5.00]	4.00 [3.00, 5.00]	3.00 [2.00, 4.00]	0.017*	4.00 [3.00, 5.00]	4.00 [3.00, 5.00]	3.00 [2.00, 4.00]	0.012*
90-day mRS (median[IQR])	3.00 [2.00, 5.00]	3.00 [2.00, 6.00]	2.00 [1.00, 4.00]	0.015*	3.00 [2.00, 5.00]	3.00 [2.00, 5.00]	2.00 [1.00, 4.00]	0.011*
Primary outcome								
90-day mRS score of 0–2, *n* (%)	61 (47.3)	26 (37.1)	35 (59.3)	0.019*	62 (44.0)	26 (33.8)	36 (56.2)	0.012*
Secondary outcomes								
90-day mortality, *n* (%)	28 (21.7)	19 (27.1)	9 (15.3)	0.156	28 (19.9)	19 (24.7)	9 (14.1)	0.174
∆mRS, (median [IQR])	2.00 [0.00, 3.00]	1.00 [−1.00, 3.00]	2.00 [1.00, 3.00]	0.060	1.00 [0.00, 3.00]	1.00 [−1.00, 3.00]	2.00 [1.00, 3.00]	0.029*
Enlargement of cerebral hyperdensity, *n* (%)	13 (10.1)	8 (11.4)	5 (8.5)	0.794	14 (9.9)	9 (11.7)	5 (7.8)	0.629
24-h hyperdensity (ECASS III)				0.149				0.022*
None	50 (38.8)	22 (31.4)	28 (47.5)		56 (39.7)	24 (31.2)	32 (50.0)	
HI1	33 (25.6)	17 (24.3)	16 (27.1)		34 (24.1)	17 (22.1)	17 (26.6)	
HI2	34 (26.4)	24 (34.3)	10 (16.9)		39 (27.7)	29 (37.7)	10 (15.6)	
PH1	4 (3.1)	2 (2.9)	2 (3.4)		4 (2.8)	2 (2.6)	2 (3.1)	
PH2	2 (1.6)	1 (1.4)	1 (1.7)		2 (1.4)	1 (1.3)	1 (1.6)	
Not performed	6 (4.7)	4 (5.7)	2 (3.4)		6 (4.3)	4 (5.2)	2 (3.1)	

*p < 0.05; **p < 0.01.

### Preliminary analysis by EAT status

As shown in Table 1, the NIHSS score before MT was lower in the EAT group (median 15 [IQR 11.5–18] vs. 18 [14–21], *p* = 0.006), whereas the NIHSS score after MT did not differ between the groups (median 11 [6–16] vs. 12 [10–16], *p* = 0.275). The immediate PCHD grade HI1 was more frequent in the EAT group (22/59 [37.3%] vs. 13/70 [18.6%], *p* = 0.029) and was associated with the outcome, as a favorable outcome occurred in 25/36 (69.4%) of patients with HI1 vs. 37/105 (35.2%) with HI2 (*p* = 0.001).

Regarding the primary outcome, a favorable outcome was achieved more frequently in the EAT group (59.3% vs. 37.1%, *p* = 0.019; Figures 2A, 3). Secondary endpoints, including ∆mRS (median 2 [IQR 1–3] vs. 1 [−1–3], *p* = 0.060), 90-day mortality (15.3% vs. 27.1%, *p* = 0.156), and enlargement of cerebral hyperdensity (8.5% vs. 11.4%, *p* = 0.794), did not differ significantly between the EAT and non-EAT groups. After imputation of missing mRS data, the primary outcome remained consistent, whereas a statistically significant difference in the secondary endpoint of ∆mRS scores was observed (median 2 [IQR 1–3] vs. 1 [−1–3], *p* = 0.029). A persistent hyperdensity on the 24-h CT was present in 79 of 135 evaluable patients (58.5%), with ECASS III morphology graded as HI1 34, HI2 39, PH1 4, and PH2 2 (Figure 2C), and was less frequent in the EAT group on unadjusted analysis (48.4% vs. 67.1%, *p* = 0.043). Baseline characteristics according to 90-day outcomes and 24-h hyperdensity are provided in Supplementary Tables S1, S2.

**Figure 2:**
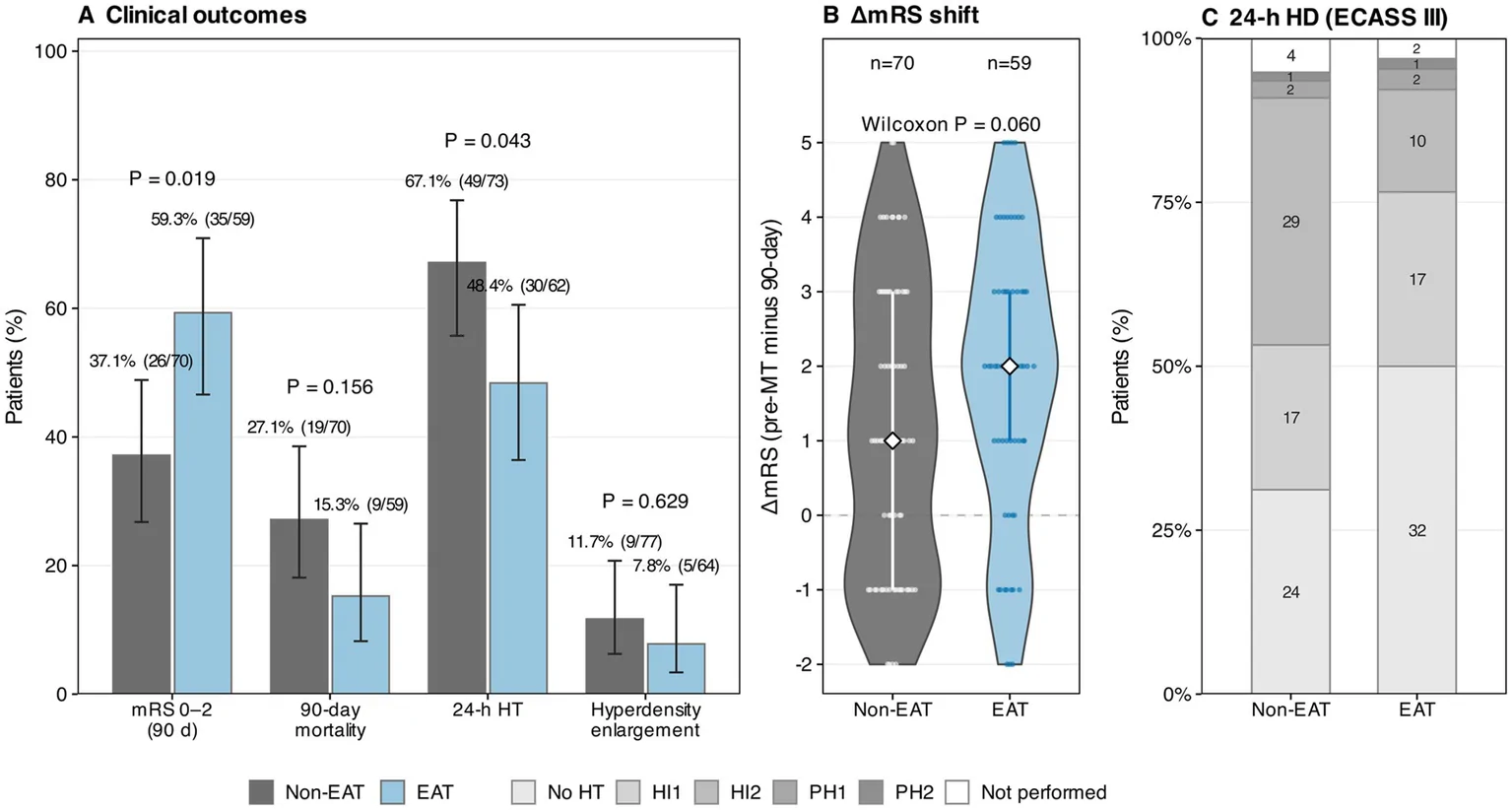
Functional and imaging outcomes according to early antithrombotic therapy. Functional outcomes were analyzed in the 129 patients with complete 90-day follow-up. Imaging variables did not depend on the 90-day mRS scores and are reported for all evaluable cases (135 had an interpretable 24-h follow-up CT; all 141 were assessed for hyperdensity enlargement). Dark gray bars, non-EAT; light blue bars, EAT; error bars, 95% Wilson CIs. Panel A gives the event rates for 90-day mRS 0–2, 90-day mortality, 24-h hyperdensity, and hyperdensity enlargement. Panel B shows the ΔmRS (pre-procedure mRS scores minus 90-day mRS scores) as a violin plot with individual observations. Panel C shows the ECASS III grade distribution of 24-h hyperdensity. Between-group comparisons used Yates’ chi-squared test **(A)**, the Mann–Whitney U test **(B)**, and Fisher’s exact test **(C)**; all *p*-values are unadjusted. EAT, early antithrombotic therapy; HD, hyperdensity; mRS, modified Rankin Scale; CT, computed tomography; ECASS, European Cooperative Acute Stroke Study.

**Figure 3:**
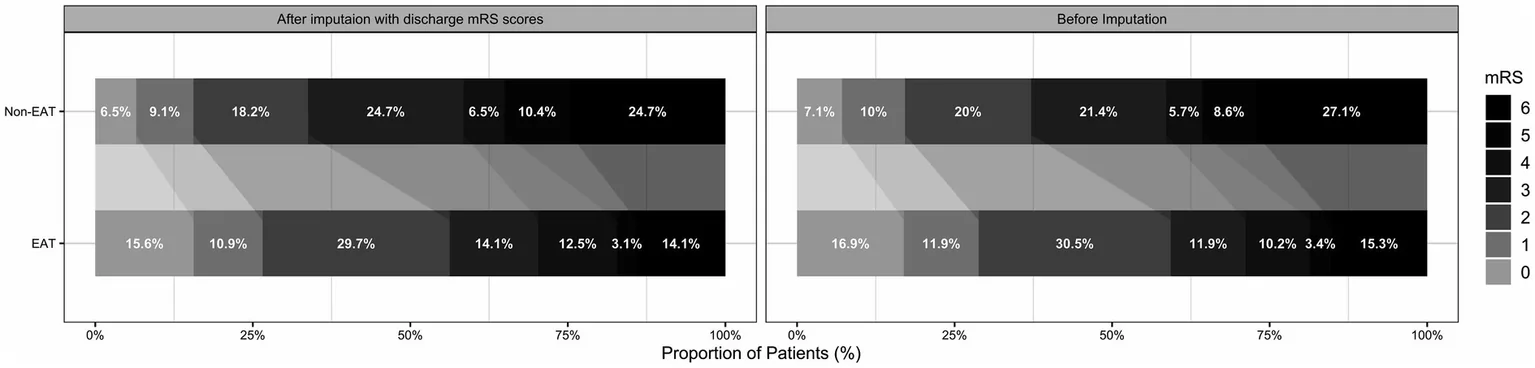
The distribution of the 90-day functional outcome grouped by early antithrombotic therapy (EAT). mRS, modified Rankin Scale.

### Univariate and multivariate logistic regression analyses of favorable outcomes

Univariate analysis stratified by favorable outcomes, regardless of imputation status, consistently indicated that EAT was associated with favorable outcomes. Based on the unadjusted absolute difference in favorable outcome rates (22.2 percentage points in complete cases and 22.5 percentage points after imputation), the corresponding descriptive numbers needed to treat (NNT) were approximately 4.5 in complete cases and 4.4 after imputation. These estimates are descriptive only and should not be interpreted as causal treatment effect. IVT before MT also showed a similar association with favorable outcomes. In contrast, a higher NIHSS score before MT, low-density lesions on CT after MT, a greater number of retrieval attempts, and early brain edema were statistically associated with unfavorable outcomes (Supplementary Table S1).

A multivariable logistic regression analysis was performed to further explore the association between EAT and favorable outcomes. EAT remained significantly associated with favorable outcomes across all models (Table 2). Model 1, adjusted for age and sex, provided odds ratios (ORs) of 2.47 (95% CI 1.20–5.11, *p* = 0.015), 2.56 (1.28–5.12, *p* = 0.008), and 2.61 (1.27–5.36, *p* = 0.009) in complete cases, after discharge imputation, and after multiple imputation, respectively. In the fully adjusted Model 2, the adjusted OR was 2.72 (1.02–7.20, *p* = 0.045) in complete cases, 2.67 (1.05–6.78, *p* = 0.040) after discharge imputation, and 2.94 (1.12–7.71, *p* = 0.028) after multiple imputation. Model 3, in which the NIHSS score before MT was replaced by the NIHSS score after MT, yielded consistent results (OR 3.68 [95% CI 1.32–10.30], 4.19 [1.51–11.60], and 3.69 [1.35–10.08]; all *p* ≤ 0.013) and was restricted to patients with a recorded NIHSS score after MT (*n* = 117 complete cases; *n* = 127 in imputed analyses). Complete covariate-level estimates for the primary outcome are provided in Supplementary Table S3.

**Table 2:** Multivariable logistic regression analysis of early antithrombotic therapy on clinical outcomes.

Outcome	Analysis before imputation			After imputing with discharge mRS scores			After multiple imputation		
	*n*	OR (95% CI)	*p*	*n*	OR (95% CI)	*p*	*n*	OR (95% CI)	*p*
Primary outcome									
90-day mRS score of 0–2									
Adjusted by Model 1	129	2.469 (1.195–5.105)	0.015	141	2.556 (1.277–5.116)	0.008	141	2.683 (1.307–5.506)	0.007
Adjusted by Model 2	129	2.715 (1.024–7.197)	0.045	141	2.665 (1.048–6.777)	0.040	141	2.939 (1.120–7.711)	0.028
Adjusted by Model 3	117	3.682 (1.316–10.302)	0.013	127	4.186 (1.511–11.597)	0.006	127	3.685 (1.347–10.079)	0.011
Secondary outcome									
ΔmRS ordinal shift									
Adjusted by Model 1	129	1.772 (0.942–3.335)	0.076	141	1.882 (1.035–3.423)	0.038	141	2.014 (1.098–3.696)	0.024
Adjusted by Model 2	129	2.049 (1.036–4.052)	0.039	141	1.919 (1.006–3.660)	0.048	141	2.243 (1.148–4.379)	0.018
Adjusted by Model 3	117	2.054 (1.014–4.159)	0.046	127	1.997 (1.010–3.948)	0.047	127	2.115 (1.060–4.221)	0.034
24-h hyperdensity (any)									
Adjusted by Model 1	135	0.462 (0.229–0.936)	0.032	—	—	—	141	0.438 (0.218–0.880)	0.020
Adjusted by Model 2	135	0.586 (0.245–1.402)	0.230	—	—	—	141	0.582 (0.243–1.393)	0.224
Adjusted by Model 3	123	0.617 (0.247–1.539)	0.301	—	—	—	127	0.616 (0.247–1.534)	0.298

*p < 0.05; **p < 0.01. Model 1 was adjusted for age and sex. Model 2 was further adjusted for low density on CT after MT, early brain edema, intravenous thrombolysis, dyslipidemia, NIHSS score before MT, number of retrieval attempts, and post-procedure ECASS classification (HI2 vs. HI1). Model 3 replaced the NIHSS score before MT with the NIHSS score after MT. Multiple imputation by chained equations (20 imputed datasets, Rubin’s rules). Only 90-day mRS scores and 24-h hyperdensity were imputed; ΔmRS scores were recalculated from imputed 90-day mRS scores. 24-h hyperdensity did not undergo discharge imputation as it is independent of 90-day mRS scores. mRS, modified Rankin Scale.

### Secondary outcomes

For the ordinal mRS shift (∆mRS), EAT was associated with a greater shift toward improvement after full adjustment (Model 2: OR = 2.05 [95% CI 1.04–4.05], *p* = 0.039 in complete cases; OR = 1.92 after discharge imputation, and OR = 2.24 after multiple imputation; Table 2; Figures 2B,3; Supplementary Table S5).

The unadjusted between-group difference in 24-h hyperdensity was significant in the minimally adjusted Model 1 (OR = 0.46 [95% CI 0.23–0.94], *p* = 0.032) but was no longer present after full adjustment (Model 2: OR = 0.59 [95% CI 0.25–1.40], *p* = 0.230; multiple imputation OR = 0.58 [0.24–1.39], *p* = 0.224; Table 2; Supplementary Table S4).

### Subgroup analysis

Subgroup analyses, stratified by age (≥80 years), sex, atrial fibrillation, IVT before MT, residual stenosis, and LAA as the cause of stroke, exhibited consistent associations across subgroups, and no significant interaction effects were identified (Figure 4, with event counts shown beneath each panel). These prespecified exploratory subgroup analyses are presented as hypothesis-generating. Point estimates were directionally consistent across strata, but estimates were imprecise, particularly in the smallest strata.

**Figure 4:**
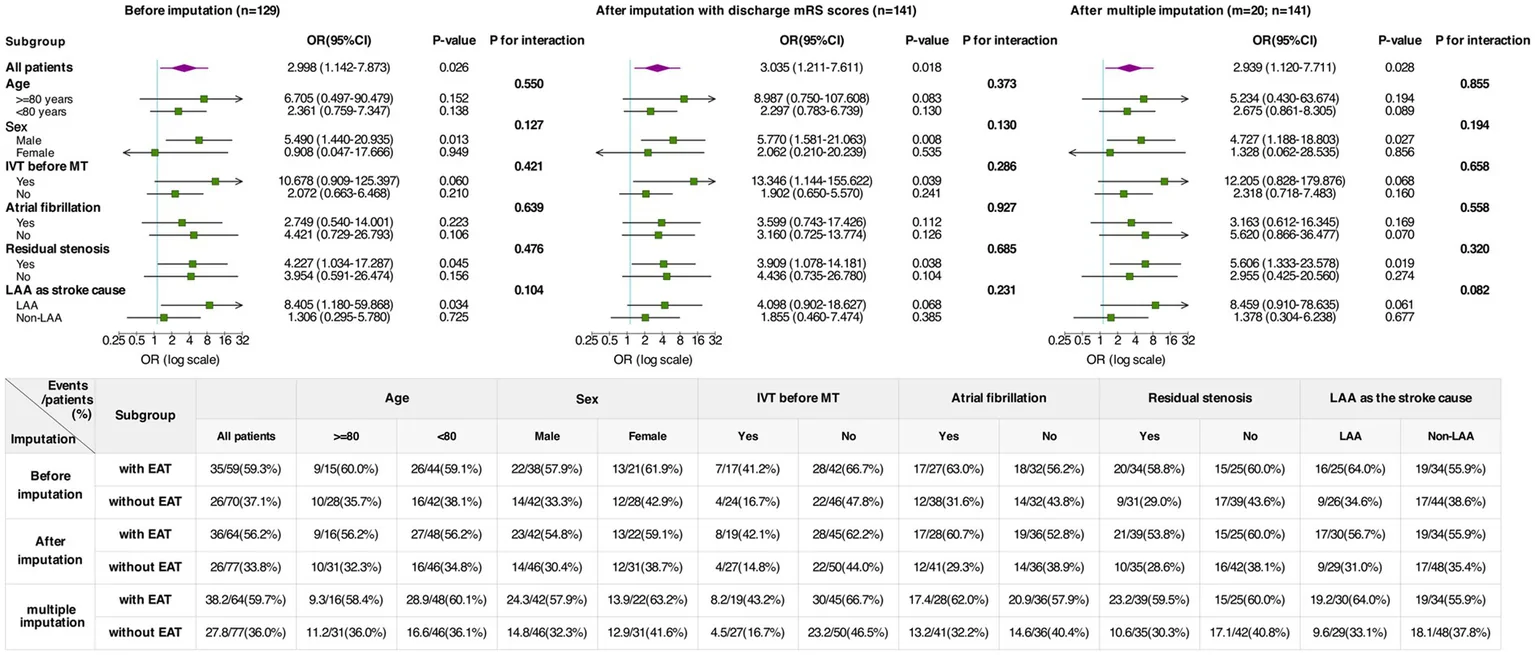
Prespecified exploratory subgroup analyses of early antithrombotic therapy for 90-day favorable functional outcomes (mRS score of 0–2). Adjusted odds ratios (ORs, 95% CIs) for 90-day favorable functional outcomes (modified Rankin Scale [mRS] score of 0–2) with early antithrombotic therapy (EAT) are shown across prespecified subgroups in the complete-case (*n* = 129), discharge-mRS imputation (*n* = 141), and multiple imputation (MICE, m = 20; *n* = 141) analyses. Squares indicate subgroup-specific ORs, and diamonds indicate the overall OR; *p*-values for interaction are from the EAT-by-subgroup term in logistic models adjusted for age, sex, low density on CT after mechanical thrombectomy, early brain edema, intravenous thrombolysis, dyslipidemia, NIHSS before MT, and number of retrieval attempts, with the stratifying variable omitted in the corresponding subgroup; the MICE models additionally included post-procedural PCHD type (ECASS III). The table below reports events/patients (%) with and without EAT. CI, confidence interval; EAT, early antithrombotic therapy; ECASS, European Cooperative Acute Stroke Study; IVT, intravenous thrombolysis; LAA, large artery atherosclerosis; MICE, multiple imputation by chained equations; mRS, modified Rankin Scale; MT, mechanical thrombectomy; NIHSS, National Institutes of Health Stroke Scale; OR, odds ratio; PCHD, postinterventional cerebral hyperdensity.

## Discussion

Compared with hyperdensity on a CT scan of the head, PCHD may help identify patients in whom EAT is associated with better outcomes. However, the concept of PCHD has not yet garnered sufficient attention. Our previous research has shown that anterior circulation stroke patients with PCHD who received EAT exhibited a lower incidence of ND. In this retrospective cohort study, after adjusting for more confounding factors, we found that anterior circulation stroke patients with PCHD who received EAT had better functional outcomes at 90 days. Moreover, the consistency of this result was preliminarily revealed in the sensitivity and subgroup analyses.

Previous studies have shown that the early initiation of specific antithrombotic therapy regimens may yield greater benefits in certain stroke patients (17–19). Compared to other stroke patients, those with PCHD often experience more severe disruption of the blood–brain barrier (BBB) (20), which makes the decision to initiate EAT challenging for clinicians. In clinical practice, we observe that patients with significant HT resulting from cerebral venous sinus thrombosis can experience gradual absorption of the HT after receiving anticoagulation treatment, which may be attributed to the difference between BBB disruption and vascular rupture-induced bleeding (11, 12). Additionally, previous studies have shown the safety of antithrombotic therapy in patients with asymptomatic hemorrhage (14, 15). Similarly, our results suggested that EAT was associated with more favorable functional outcomes in patients with PCHD, a concept that specifically excludes hyperdensity with a space-occupying effect or HT outside the brain parenchyma.

In our study, the type of antithrombotic drugs was selected based on the patient’s etiology. Furthermore, the subgroup analysis revealed that the LAA group had a greater absolute difference in favorable outcome rates than the non-LAA group, which is consistent with previous findings that atherosclerotic lesions are more prone to early ND (21, 22). Additionally, we found that older age, male sex, and IVT before MT might be associated with greater benefits from EAT. However, these subgroups did not exhibit a statistically significant interaction between EAT and 90-day favorable outcomes, which may be due to the limited sample size in the subgroup analysis. These findings should be further evaluated in future studies with larger sample sizes.

As to why EAT might benefit patients with PCHD, we speculate that there may be two main reasons. First, a recent meta-analysis indicated that within 24 h post-MT, 3–9% of AIS patients experience re-occlusion (23). EAT can reduce the risk of re-occlusion. Second, following a successful thrombectomy, 3–42.5% of patients experience the no-reflow phenomenon (24, 25), which may be attributed to increased blood viscosity and slow blood flow resulting from stagnant blood components (26). This can lead to microcirculatory disturbances, such as capillary thrombosis (6), and also provides a theoretical basis for adjunctive intra-arterial thrombolysis after angiographically successful thrombectomy (27).

Dual-energy CT can distinguish HT from contrast extravasation, with a diagnostic accuracy ranging from 33 to 100% (28). Although our center’s dual-source CT (Siemens SOMATOM Force) can differentiate contrast extravasation from HT without a space-occupying effect, we did not perform immediate dual-energy CT scans on post-MT patients due to the distance between the dual-source CT and the operating room and wards. However, we currently believe that, in the context of EAT, distinguishing between contrast extravasation and HT without a space-occupying effect may be less critical for clinical decision-making, as some studies have also shown the safety of EAT in patients with HT (14, 15). Because the immediate post-MT CT was acquired on a single-energy scanner, we could not reliably distinguish hemorrhagic transformation from contrast extravasation. We therefore described the 24-h follow-up CT by ECASS III morphology as a hyperdensity outcome and did not report the proportion of presumed contrast staining.

Our study offers several strengths and limitations that are important to consider. First, to the best of our knowledge, this is the first study to specifically investigate the association between EAT and 90-day functional outcomes in patients with PCHD. This focus provides new insights into the potential role of EAT in this specific patient population. Second, our study included data from before the combination of our postoperative management teams. Because different attendings had varying treatment philosophies regarding EAT in AIS patients with PCHD, the EAT and non-EAT groups were assigned in a manner that approximated randomization, thereby reducing selection bias and ensuring that baseline data, except for NIHSS scores before MT, did not show significant differences between the groups. The observed differences in NIHSS scores may be due to the tendency of attending physicians who administered EAT to admit patients with higher NIHSS scores. To further explore these associations, we performed multivariable analyses for each outcome, together with sensitivity and subgroup analyses to assess the robustness and internal consistency of our results.

However, our study also has limitations. This was a retrospective single-center study with a limited sample size, and we cannot rule out residual confounding despite multivariable adjustment. The immediate post-MT CT was not acquired on a dual-energy scanner, so contrast extravasation could not be reliably distinguished from hemorrhagic transformation; the 24-h findings are therefore reported as hyperdensity graded by ECASS III morphology. We did not systematically record PCHD volume or anatomical location, which should be addressed in future multicenter studies. Multiple imputation was used to address missing 90-day mRS data, but residual bias due to missing imaging data cannot be ruled out.

## Conclusion

EAT is associated with improved 90-day functional outcomes in AIS patients with PCHD. These findings warrant further investigation in larger prospective studies.

## Data Availability

The data that support the findings of this study are available from the corresponding author upon reasonable request. Requests to access these datasets should be directed to Jin Zheng, zhengjin22163@fudan.edu.cn.
