# Supplementary material for: Early antithrombotic therapy improves 90-day functional outcomes in patients with postinterventional cerebral hyperdensity after mechanical thrombectomy: a retrospective cohort study
Source: Front Neurol. 2026 Oct 23;17:1891162. doi: 10.3389/fneur.2026.1891162 (PMC13739585; doi:10.3389/fneur.2026.1891162)
Supplement: Supplementary file 1 [file Supplementary_file_1.DOCX]

**Supplementary material**

**Supplementary Figure**

Figure S1. Convergence and stability of the multiple imputation procedure.

**Supplementary Tables**

Table S1. Baseline characteristics by 90-day functional outcomes.

Table S2. Baseline characteristics by 24-hour hyperdensity.

Table S3. Multivariable logistic regression for favorable functional outcome.

Table S4. Full multivariable logistic regression for any 24-hour hyperdensity.

Table S5. Ordinal logistic regression (proportional odds) for ΔmRS shift toward improvement.

Figure S1. Convergence and stability of the multiple imputation procedure.

Multiple imputation by chained equations was run with 20 imputed data sets and 10 iterations to recover the 12 missing 90-day mRS scores and the 6 patients without an interpretable 24-h CT. The ordinal 90-day mRS was imputed by proportional odds regression and 24h hyperdensity by predictive mean matching, with discharge mRS and post-procedure ECASS grade as predictors. The 20 chain traces are flat from the second iteration onward (A, C); B and D compare the observed with the imputed distributions of mRS and 24h hyperdensity; and E shows the event rates staying within a narrow range across the 20 completed data sets. The imputation converged, and the estimates are not sensitive to any single imputed data set.

**Abbreviations:** mRS, modified Rankin Scale; CT, computed tomography; ECASS, European Cooperative Acute Stroke Study.

Table S1. Baseline characteristics by 90-day functional outcomes.

| Characteristic | Analysis before imputing mRS scores | | | | Analysis after imputing with discharge mRS scores | | | |
| --- | --- | --- | --- | --- | --- | --- | --- | --- |
|  | Total | Unfavorable | Favorable | P value | Total | Unfavorable | Favorable | P value |
|  | (n=129) | (n=68) | (n=61) |  | (n=141) | (n=79) | (n=62) |  |
| **Demographics** |  |  |  |  |  |  |  |  |
| Female, n(%) | 49 (38.0) | 24 ( 35.3) | 25 ( 41.0) | 0.629 | 53 (37.6) | 28 ( 35.4) | 25 ( 40.3) | 0.675 |
| Age, (median[IQR]) | 72.00 [66.00, 83.00] | 71.00 [66.75, 84.00] | 74.00 [64.00, 80.00] | 0.923 | 72.00 [66.00, 82.00] | 71.00 [66.50, 82.50] | 73.50 [63.25, 80.00] | 0.914 |
| Hypertension, n(%) | 80 (62.0) | 45 ( 66.2) | 35 ( 57.4) | 0.397 | 85 (60.3) | 49 ( 62.0) | 36 ( 58.1) | 0.761 |
| Diabetes mellitus, n(%) | 39 (30.2) | 24 ( 35.3) | 15 ( 24.6) | 0.259 | 43 (30.5) | 28 ( 35.4) | 15 ( 24.2) | 0.209 |
| Dyslipidemia, n(%) | 39 (30.2) | 15 ( 22.1) | 24 ( 39.3) | 0.052 | 43 (30.5) | 18 ( 22.8) | 25 ( 40.3) | 0.039* |
| Atrial fibrillation, n(%) | 65 (50.4) | 36 ( 52.9) | 29 ( 47.5) | 0.663 | 69 (48.9) | 40 ( 50.6) | 29 ( 46.8) | 0.775 |
| Smoking history, n(%) |  |  |  | 0.825 |  |  |  | 0.666 |
| Non-smoker | 98 (76.0) | 53 ( 77.9) | 45 ( 73.8) |  | 107 (75.9) | 62 ( 78.5) | 45 ( 72.6) |  |
| Former smoker | 9 ( 7.0) | 4 ( 5.9) | 5 ( 8.2) |  | 9 ( 6.4) | 4 ( 5.1) | 5 ( 8.1) |  |
| Current smoker | 22 (17.1) | 11 ( 16.2) | 11 ( 18.0) |  | 25 (17.7) | 13 ( 16.5) | 12 ( 19.4) |  |
| Premorbid mRS, n(%) |  |  |  | 0.553 |  |  |  | 0.657 |
| 0 | 81 (62.8) | 45 ( 66.2) | 36 ( 59.0) |  | 90 (63.8) | 53 ( 67.1) | 37 ( 59.7) |  |
| 1 | 31 (24.0) | 16 ( 23.5) | 15 ( 24.6) |  | 31 (22.0) | 16 ( 20.3) | 15 ( 24.2) |  |
| 2 | 17 (13.2) | 7 ( 10.3) | 10 ( 16.4) |  | 20 (14.2) | 10 ( 12.7) | 10 ( 16.1) |  |
| **Clinical characteristics** |  |  |  |  |  |  |  |  |
| NIHSS score before MT, (median[IQR]) | 16.00 [12.00, 20.00] | 18.00 [14.00, 20.25] | 15.00 [11.00, 18.00] | 0.008** | 16.00 [12.00, 20.00] | 18.00 [14.00, 20.50] | 15.00 [11.25, 18.75] | 0.007** |
| NIHSS after MT, (median[IQR]) | 12.00 [7.00, 16.00] | 14.00 [10.00, 18.00] | 10.00 [5.00, 13.00] | 0.001** | 12.00 [7.50, 17.00] | 14.00 [10.00, 18.00] | 10.00 [5.00, 14.00] | 0.001** |
| mRS scores before MT, (median[IQR]) | 5.00 [4.00, 5.00] | 5.00 [4.00, 5.00] | 5.00 [4.00, 5.00] | 0.071 | 5.00 [4.00, 5.00] | 5.00 [4.00, 5.00] | 5.00 [4.00, 5.00] | 0.069 |
| PCHD type (ECASS III), n(%) |  |  |  | 0.006** |  |  |  | 0.001** |
| HI1 | 35 (27.1) | 11 ( 16.2) | 24 ( 39.3) |  | 36 (25.5) | 11 ( 13.9) | 25 ( 40.3) |  |
| HI2 | 94 (72.9) | 57 ( 83.8) | 37 ( 60.7) |  | 105 (74.5) | 68 ( 86.1) | 37 ( 59.7) |  |
| IVT before MT, n(%) | 41 (31.8) | 30 ( 44.1) | 11 ( 18.0) | 0.003** | 46 (32.6) | 34 ( 43.0) | 12 ( 19.4) | 0.005** |
| Residual stenosis, n(%) | 65 (50.4) | 36 ( 52.9) | 29 ( 47.5) | 0.663 | 74 (52.5) | 43 ( 54.4) | 31 ( 50.0) | 0.724 |
| Stent implation, n(%) | 6 ( 4.7) | 4 ( 5.9) | 2 ( 3.3) | 0.778 | 6 ( 4.3) | 4 ( 5.1) | 2 ( 3.2) | 0.907 |
| Low density on CT after MT, n(%) |  |  |  | <0.001 |  |  |  | <0.001 |
| Without low density on CT | 36 (27.9) | 10 ( 14.7) | 26 ( 42.6) |  | 38 (27.0) | 12 ( 15.2) | 26 ( 41.9) |  |
| Low density < 1/3 vascular territory | 70 (54.3) | 39 ( 57.4) | 31 ( 50.8) |  | 75 (53.2) | 43 ( 54.4) | 32 ( 51.6) |  |
| Low density > 1/3 vascular territory | 23 (17.8) | 19 ( 27.9) | 4 ( 6.6) |  | 28 (19.9) | 24 ( 30.4) | 4 ( 6.5) |  |
| Initial occlusion site, n(%) |  |  |  | 0.420 |  |  |  | 0.194 |
| ICA | 42 (32.6) | 26 ( 38.2) | 16 ( 26.2) |  | 51 (36.2) | 34 ( 43.0) | 17 ( 27.4) |  |
| M1 | 67 (51.9) | 34 ( 50.0) | 33 ( 54.1) |  | 70 (49.6) | 37 ( 46.8) | 33 ( 53.2) |  |
| M2 | 10 ( 7.8) | 4 ( 5.9) | 6 ( 9.8) |  | 10 ( 7.1) | 4 ( 5.1) | 6 ( 9.7) |  |
| Tandem | 10 ( 7.8) | 4 ( 5.9) | 6 ( 9.8) |  | 10 ( 7.1) | 4 ( 5.1) | 6 ( 9.7) |  |
| MT techniques, n(%) |  |  |  | 0.458 |  |  |  | 0.376 |
| SR | 5 ( 3.9) | 4 ( 5.9) | 1 ( 1.6) |  | 6 ( 4.3) | 5 ( 6.3) | 1 ( 1.6) |  |
| CA | 6 ( 4.7) | 3 ( 4.4) | 3 ( 4.9) |  | 6 ( 4.3) | 3 ( 3.8) | 3 ( 4.8) |  |
| Combined SR and CA | 118 (91.5) | 61 ( 89.7) | 57 ( 93.4) |  | 129 (91.5) | 71 ( 89.9) | 58 ( 93.5) |  |
| TICI, n(%) |  |  |  | 0.222 |  |  |  | 0.221 |
| 0 | 2 ( 1.6) | 2 ( 2.9) | 0 ( 0.0) |  | 2 ( 1.4) | 2 ( 2.5) | 0 ( 0.0) |  |
| 1 | 5 ( 3.9) | 4 ( 5.9) | 1 ( 1.6) |  | 5 ( 3.5) | 4 ( 5.1) | 1 ( 1.6) |  |
| 2a | 8 ( 6.2) | 6 ( 8.8) | 2 ( 3.3) |  | 10 ( 7.1) | 8 ( 10.1) | 2 ( 3.2) |  |
| 2b | 35 (27.1) | 16 ( 23.5) | 19 ( 31.1) |  | 40 (28.4) | 21 ( 26.6) | 19 ( 30.6) |  |
| 3 | 79 (61.2) | 40 ( 58.8) | 39 ( 63.9) |  | 84 (59.6) | 44 ( 55.7) | 40 ( 64.5) |  |
| Stroke subtype, n(%) |  |  |  | 0.920 |  |  |  | 0.960 |
| LAA | 51 (39.5) | 26 ( 38.2) | 25 ( 41.0) |  | 59 (41.8) | 33 ( 41.8) | 26 ( 41.9) |  |
| CE | 56 (43.4) | 31 ( 45.6) | 25 ( 41.0) |  | 59 (41.8) | 34 ( 43.0) | 25 ( 40.3) |  |
| SOE | 7 ( 5.4) | 4 ( 5.9) | 3 ( 4.9) |  | 7 ( 5.0) | 4 ( 5.1) | 3 ( 4.8) |  |
| SUE | 15 (11.6) | 7 ( 10.3) | 8 ( 13.1) |  | 16 (11.3) | 8 ( 10.1) | 8 ( 12.9) |  |
| Early brain edema, n(%) | 31 (24.0) | 28 ( 41.2) | 3 ( 4.9) | <0.001 | 36 (25.5) | 33 ( 41.8) | 3 ( 4.8) | <0.001 |
| **Procedure** |  |  |  |  |  |  |  |  |
| DPT, min, (median[IQR]) | 90.00 [79.00, 121.00] | 88.00 [78.25, 121.00] | 92.00 [79.00, 120.00] | 0.889 | 92.00 [79.00, 121.00] | 89.00 [77.50, 125.00] | 92.00 [79.50, 120.00] | 0.957 |
| OFT, min, (median[IQR]) | 295.00 [202.00, 428.00] | 303.50 [221.25, 380.50] | 290.00 [200.00, 447.00] | 0.814 | 294.00 [202.00, 413.00] | 294.00 [220.50, 375.00] | 292.50 [200.00, 447.00] | 0.664 |
| Number of retrieval attempts, n(%) |  |  |  | 0.001** |  |  |  | <0.001 |
| 1 | 61 (47.3) | 23 ( 33.8) | 38 ( 62.3) |  | 63 (44.7) | 24 ( 30.4) | 39 ( 62.9) |  |
| 2 | 24 (18.6) | 12 ( 17.6) | 12 ( 19.7) |  | 28 (19.9) | 16 ( 20.3) | 12 ( 19.4) |  |
| 3 | 24 (18.6) | 15 ( 22.1) | 9 ( 14.8) |  | 27 (19.1) | 18 ( 22.8) | 9 ( 14.5) |  |
| ≥4 | 20 (15.5) | 18 ( 26.5) | 2 ( 3.3) |  | 23 (16.3) | 21 ( 26.6) | 2 ( 3.2) |  |
| **Early antithrombotic therapy, n(%)** | 59 (45.7) | 24 ( 35.3) | 35 ( 57.4) | 0.019* | 64 (45.4) | 28 ( 35.4) | 36 ( 58.1) | 0.012* |

*means P < 0.05; **means P < 0.01

Abbreviations: NIHSS, National Institutes of Health Stroke Scale; IVT, intravenous thrombolysis ; MT, mechanical thrombectomy; LAA, large-artery atherosclerosis; CE, cardioembolism; SOE, stroke of other determined etiology; SUE, stroke of undetermined etiology; SR, stent retriever; CA, contact aspiration; ICA, internal carotid artery; DPT, door-to-puncture time; OFT, onset to final TICI score time.

Table S2. Baseline characteristics by 24-hour hyperdensity.

| Characteristic | Analysis before multiple imputation | | | | Analysis after multiple imputation | | | |
| --- | --- | --- | --- | --- | --- | --- | --- | --- |
|  | Total | No 24h hyperdensity | 24h hyperdensity | P value | Total | No 24h hyperdensity | 24h hyperdensity | P value |
|  | (n=135) | (n=56) | (n=79) |  | (n=141) | (n=57) | (n=84) |  |
| **Demographics** |  |  |  |  |  |  |  |  |
| Female, n(%) | 49 (36.3) | 21 ( 37.5) | 28 (35.4) | 0.950 | 53.0 (37.6) | 21.9 (38.5) | 31.1 (37.0) | 0.861 |
| Age, (median[IQR]) | 72.00 [65.00, 81.00] | 73.00 [59.25, 84.00] | 71.00 [66.00, 80.50] | 0.904 | 72.00 [66.00, 82.00] | 72.40 [60.08, 84.03] | 72.35 [66.72, 81.17] | 0.819 |
| Hypertension, n(%) | 82 (60.7) | 33 ( 58.9) | 49 (62.0) | 0.854 | 85.0 (60.3) | 33.0 (58.0) | 52.0 (61.8) | 0.726 |
| Diabetes mellitus, n(%) | 41 (30.4) | 16 ( 28.6) | 25 (31.6) | 0.847 | 43.0 (30.5) | 16.1 (28.2) | 26.9 (32.1) | 0.710 |
| Dyslipidemia, n(%) | 43 (31.9) | 18 ( 32.1) | 25 (31.6) | 1.000 | 43.0 (30.5) | 18.0 (31.6) | 25.0 (29.8) | 0.854 |
| Atrial fibrillation, n(%) | 63 (46.7) | 23 ( 41.1) | 40 (50.6) | 0.356 | 69.0 (48.9) | 24.1 (42.3) | 44.9 (53.5) | 0.230 |
| Smoking history, n(%) |  |  |  | 0.973 |  |  |  | 1.000 |
| Non-smoker | 101 (74.8) | 42 ( 75.0) | 59 (74.7) |  | 107.0 (75.9) | 43.1 (75.6) | 63.9 (76.1) |  |
| Former smoker | 9 ( 6.7) | 4 ( 7.1) | 5 ( 6.3) |  | 9.0 (6.4) | 4.0 (7.0) | 5.0 (6.0) |  |
| Current smoker | 25 (18.5) | 10 ( 17.9) | 15 (19.0) |  | 25.0 (17.7) | 10.0 (17.5) | 15.0 (17.9) |  |
| Premorbid mRS, n(%) |  |  |  | 0.319 |  |  |  | 0.321 |
| 0 | 86 (63.7) | 32 ( 57.1) | 54 (68.4) |  | 90.0 (63.8) | 33.1 (58.1) | 56.9 (67.7) |  |
| 1 | 29 (21.5) | 13 ( 23.2) | 16 (20.3) |  | 31.0 (22.0) | 13.0 (22.8) | 18.0 (21.4) |  |
| 2 | 20 (14.8) | 11 ( 19.6) | 9 (11.4) |  | 20.0 (14.2) | 11.0 (19.3) | 9.0 (10.7) |  |
| **Clinical characteristics** |  |  |  |  |  |  |  |  |
| NIHSS score before MT, (median[IQR]) | 16.00 [12.00, 20.00] | 15.00 [12.00, 19.00] | 17.00 [13.00, 20.50] | 0.086 | 16.00 [12.00, 20.00] | 15.00 [12.00, 19.02] | 17.35 [13.00, 21.86] | 0.024* |
| NIHSS after MT, (median[IQR]) | 12.00 [7.50, 16.00] | 10.00 [6.00, 14.25] | 13.00 [10.00, 17.00] | 0.036* | 12.00 [7.50, 17.00] | 10.00 [6.00, 14.16] | 13.00 [10.00, 17.00] | 0.012* |
| mRS scores before MT, (median[IQR]) | 5.00 [4.00, 5.00] | 4.00 [4.00, 5.00] | 5.00 [4.00, 5.00] | 0.056 | 5.00 [4.00, 5.00] | 4.00 [4.00, 5.00] | 5.00 [4.00, 5.00] | 0.022* |
| PCHD type (ECASS III), n(%) |  |  |  | <0.001 |  |  |  | <0.001 |
| HI1 | 35 (25.9) | 27 ( 48.2) | 8 (10.1) |  | 36.0 (25.5) | 27.9 (48.9) | 8.2 (9.7) |  |
| HI2 | 100 (74.1) | 29 ( 51.8) | 71 (89.9) |  | 105.0 (74.5) | 29.2 (51.3) | 75.8 (90.2) |  |
| IVT before MT, n(%) | 43 (31.9) | 15 ( 26.8) | 28 (35.4) | 0.381 | 46.0 (32.6) | 15.2 (26.6) | 30.9 (36.7) | 0.205 |
| Residual stenosis, n(%) | 72 (53.3) | 26 ( 46.4) | 46 (58.2) | 0.238 | 74.0 (52.5) | 26.9 (47.3) | 47.0 (56.0) | 0.391 |
| Stent implation, n(%) | 6 ( 4.4) | 3 ( 5.4) | 3 ( 3.8) | 0.992 | 6.0 (4.3) | 3.0 (5.3) | 3.0 (3.6) | 0.686 |
| Low density on CT after MT, n(%) |  |  |  | 0.006** |  |  |  | 0.002** |
| Without low density on CT | 37 (27.4) | 23 ( 41.1) | 14 (17.7) |  | 38.0 (27.0) | 23.9 (41.8) | 14.2 (16.8) |  |
| Low density < 1/3 vascular territory | 73 (54.1) | 27 ( 48.2) | 46 (58.2) |  | 75.0 (53.2) | 27.1 (47.5) | 48.0 (57.1) |  |
| Low density > 1/3 vascular territory | 25 (18.5) | 6 ( 10.7) | 19 (24.1) |  | 28.0 (19.9) | 6.2 (10.9) | 21.8 (26.0) |  |
| Initial occlusion site, n(%) |  |  |  | 0.473 |  |  |  | 0.521 |
| ICA | 48 (35.6) | 19 ( 33.9) | 29 (36.7) |  | 51.0 (36.2) | 19.2 (33.7) | 31.8 (37.9) |  |
| M1 | 68 (50.4) | 26 ( 46.4) | 42 (53.2) |  | 70.0 (49.6) | 26.9 (47.2) | 43.1 (51.3) |  |
| M2 | 10 ( 7.4) | 6 ( 10.7) | 4 ( 5.1) |  | 10.0 (7.1) | 6.0 (10.5) | 4.0 (4.8) |  |
| Tandem | 9 ( 6.7) | 5 ( 8.9) | 4 ( 5.1) |  | 10.0 (7.1) | 5.0 (8.8) | 5.0 (6.0) |  |
| MT techniques, n(%) |  |  |  | 0.645 |  |  |  | 0.902 |
| SR | 6 ( 4.4) | 2 ( 3.6) | 4 ( 5.1) |  | 6.0 (4.3) | 2.0 (3.5) | 4.0 (4.8) |  |
| CA | 5 ( 3.7) | 3 ( 5.4) | 2 ( 2.5) |  | 6.0 (4.3) | 3.0 (5.3) | 3.0 (3.6) |  |
| Combined SR and CA | 124 (91.9) | 51 ( 91.1) | 73 (92.4) |  | 129.0 (91.5) | 52.1 (91.4) | 76.9 (91.5) |  |
| TICI, n(%) |  |  |  | 0.032* |  |  |  | 0.027* |
| 0 | 2 ( 1.5) | 0 ( 0.0) | 2 ( 2.5) |  | 2.0 (1.4) | 0.0 (0.0) | 2.0 (2.4) |  |
| 1 | 5 ( 3.7) | 2 ( 3.6) | 3 ( 3.8) |  | 5.0 (3.5) | 2.0 (3.5) | 3.0 (3.6) |  |
| 2a | 9 ( 6.7) | 1 ( 1.8) | 8 (10.1) |  | 10.0 (7.1) | 1.1 (1.8) | 8.9 (10.7) |  |
| 2b | 40 (29.6) | 12 ( 21.4) | 28 (35.4) |  | 40.0 (28.4) | 12.0 (21.1) | 28.0 (33.3) |  |
| 3 | 79 (58.5) | 41 ( 73.2) | 38 (48.1) |  | 84.0 (59.6) | 42.0 (73.8) | 42.0 (49.9) |  |
| Stroke subtype, n(%) |  |  |  | 0.380 |  |  |  | 0.341 |
| LAA | 58 (43.0) | 23 ( 41.1) | 35 (44.3) |  | 59.0 (41.8) | 23.9 (41.8) | 35.1 (41.8) |  |
| CE | 55 (40.7) | 21 ( 37.5) | 34 (43.0) |  | 59.0 (41.8) | 21.2 (37.3) | 37.8 (44.9) |  |
| SOE | 7 ( 5.2) | 5 ( 8.9) | 2 ( 2.5) |  | 7.0 (5.0) | 5.0 (8.8) | 2.0 (2.4) |  |
| SUE | 15 (11.1) | 7 ( 12.5) | 8 (10.1) |  | 16.0 (11.3) | 7.0 (12.3) | 9.0 (10.7) |  |
| Early brain edema, n(%) | 31 (23.0) | 5 ( 8.9) | 26 (32.9) | 0.002** | 36.0 (25.5) | 5.2 (9.2) | 30.8 (36.6) | <0.001 |
| **Procedure** |  |  |  |  |  |  |  |  |
| DPT, min, (median[IQR]) | 90.00 [76.50, 121.00] | 93.00 [76.00, 122.25] | 88.00 [78.00, 120.50] | 0.590 | 92.00 [79.00, 121.00] | 93.95 [76.15, 125.12] | 89.50 [79.72, 120.96] | 0.670 |
| OFT, min, (median[IQR]) | 290.00 [200.50, 412.00] | 305.00 [226.25, 473.00] | 271.00 [197.50, 375.00] | 0.375 | 294.00 [202.00, 413.00] | 307.85 [229.62, 484.50] | 271.50 [200.00, 381.96] | 0.346 |
| Number of retrieval attempts, n(%) |  |  |  | 0.042* |  |  |  | 0.022* |
| 1 | 61 (45.2) | 30 ( 53.6) | 31 (39.2) |  | 63.0 (44.7) | 30.1 (52.7) | 33.0 (39.2) |  |
| 2 | 28 (20.7) | 13 ( 23.2) | 15 (19.0) |  | 28.0 (19.9) | 13.0 (22.8) | 15.0 (17.9) |  |
| 3 | 25 (18.5) | 10 ( 17.9) | 15 (19.0) |  | 27.0 (19.1) | 10.9 (19.1) | 16.1 (19.2) |  |
| ≥4 | 21 (15.6) | 3 ( 5.4) | 18 (22.8) |  | 23.0 (16.3) | 3.1 (5.5) | 19.9 (23.6) |  |
| **Early antithrombotic therapy, n(%)** | 62 (45.9) | 32 ( 57.1) | 30 (38.0) | 0.043* | 64.0 (45.4) | 33.0 (57.9) | 31.0 (36.9) | 0.016* |

Table S3.Multivariable logistic regression for favorable functional outcome.

| **Variable** | **Before imputing mRS scores (n=129)** | | | | | | **After imputing with discharge mRS scores (n=141)** | | | | | | **After multiple imputation (n=141)** | | | | | |
| --- | --- | --- | --- | --- | --- | --- | --- | --- | --- | --- | --- | --- | --- | --- | --- | --- | --- | --- |
|  | **Model 1** | | **Model 2** | | **Model 3** | | **Model 1** | | **Model 2** | | **Model 3** | | **Model 1** | | **Model 2** | | **Model 3** | |
|  | **OR (95% CI)** | **P** | **OR (95% CI)** | **P** | **OR (95% CI)** | **P** | **OR (95% CI)** | **P** | **OR (95% CI)** | **P** | **OR (95% CI)** | **P** | **OR (95% CI)** | **P** | **OR (95% CI)** | **P** | **OR (95% CI)** | **P** |
| Early antithrombotic treatment | 2.469 (1.195-5.105) | 0.015* | 2.715 (1.024-7.197) | 0.045* | 3.682 (1.316-10.302) | 0.013* | 2.556 (1.277-5.116) | 0.008** | 2.665 (1.048-6.777) | 0.040* | 4.186 (1.511-11.597) | 0.006** | 2.683 (1.307-5.506) | 0.007** | 2.939 (1.120-7.711) | 0.028* | 3.685 (1.347-10.079) | 0.011* |
| Male | 1.407 (0.630-3.145) | 0.405 | 1.370 (0.480-3.916) | 0.556 | 1.262 (0.425-3.749) | 0.676 | 1.353 (0.633-2.890) | 0.435 | 1.109 (0.407-3.021) | 0.840 | 1.114 (0.385-3.222) | 0.842 | 1.408 (0.645-3.071) | 0.390 | 1.204 (0.431-3.365) | 0.724 | 1.188 (0.411-3.434) | 0.750 |
| Age | 0.996 (0.968-1.025) | 0.798 | 1.013 (0.972-1.056) | 0.541 | 1.023 (0.980-1.069) | 0.296 | 0.998 (0.972-1.025) | 0.877 | 1.012 (0.973-1.053) | 0.545 | 1.022 (0.981-1.065) | 0.291 | 0.998 (0.971-1.027) | 0.904 | 1.011 (0.970-1.054) | 0.598 | 1.018 (0.975-1.063) | 0.409 |
| NIHSS score before MT | - | - | 0.943 (0.863-1.031) | 0.197 | - | - | - | - | 0.946 (0.870-1.030) | 0.200 | - | - | - | - | 0.959 (0.879-1.045) | 0.340 | - | - |
| NIHSS score after MT (post-procedure) | - | - | - | - | 0.893 (0.807-0.990) | 0.031* | - | - | - | - | 0.908 (0.825-1.000) | 0.049* | - | - | - | - | 0.911 (0.827-1.003) | 0.057 |
| IVT before MT | - | - | 0.283 (0.098-0.815) | 0.019* | 0.184 (0.055-0.620) | 0.006** | - | - | 0.302 (0.107-0.851) | 0.024* | 0.213 (0.067-0.685) | 0.009** | - | - | 0.298 (0.105-0.843) | 0.022* | 0.225 (0.069-0.733) | 0.013* |
| Dyslipidemia | - | - | 2.051 (0.750-5.606) | 0.161 | 2.298 (0.804-6.568) | 0.121 | - | - | 2.019 (0.770-5.294) | 0.153 | 2.702 (0.952-7.672) | 0.062 | - | - | 1.901 (0.703-5.139) | 0.205 | 2.107 (0.745-5.960) | 0.160 |
| Early brain edema | - | - | 0.364 (0.080-1.656) | 0.191 | 0.706 (0.133-3.756) | 0.683 | - | - | 0.327 (0.073-1.456) | 0.142 | 0.598 (0.119-3.011) | 0.533 | - | - | 0.396 (0.091-1.714) | 0.215 | 0.630 (0.123-3.226) | 0.579 |
| PCHD type (ECASS III, HI2 vs HI1) | - | - | 0.365 (0.123-1.083) | 0.069 | 0.352 (0.111-1.114) | 0.076 | - | - | 0.330 (0.114-0.954) | 0.041* | 0.315 (0.101-0.980) | 0.046* | - | - | 0.394 (0.134-1.156) | 0.090 | 0.374 (0.120-1.167) | 0.090 |
| Low density on CT after MT |  |  |  |  |  |  |  |  |  |  |  |  |  |  |  |  |  |  |
| Without low density on CT | - | - | Reference |  | Reference |  | - | - | Reference |  | Reference |  | - | - | Reference |  | Reference |  |
| Low density < 1/3 vascular territory | - | - | 0.416 (0.141-1.229) | 0.112 | 0.583 (0.194-1.755) | 0.337 | - | - | 0.530 (0.191-1.476) | 0.225 | 0.664 (0.224-1.962) | 0.458 | - | - | 0.420 (0.144-1.223) | 0.112 | 0.605 (0.203-1.806) | 0.368 |
| Low density > 1/3 vascular territory | - | - | 0.182 (0.034-0.984) | 0.048* | 0.277 (0.047-1.648) | 0.158 | - | - | 0.218 (0.042-1.132) | 0.070 | 0.280 (0.048-1.633) | 0.157 | - | - | 0.218 (0.040-1.190) | 0.079 | 0.312 (0.052-1.876) | 0.203 |
| Number of retrieval attempts |  |  |  |  |  |  |  |  |  |  |  |  |  |  |  |  |  |  |
| 1 | - | - | Reference |  | Reference |  | - | - | Reference |  | Reference |  | - | - | Reference |  | Reference |  |
| 2 | - | - | 0.656 (0.193-2.227) | 0.499 | 0.655 (0.181-2.365) | 0.518 | - | - | 0.483 (0.150-1.555) | 0.223 | 0.557 (0.160-1.940) | 0.358 | - | - | 0.657 (0.200-2.161) | 0.490 | 0.658 (0.189-2.296) | 0.512 |
| 3 | - | - | 0.198 (0.057-0.693) | 0.011* | 0.160 (0.043-0.598) | 0.006** | - | - | 0.196 (0.058-0.662) | 0.009** | 0.152 (0.041-0.560) | 0.005** | - | - | 0.210 (0.060-0.730) | 0.014* | 0.160 (0.043-0.590) | 0.006** |
| ≥4 | - | - | 0.159 (0.026-0.953) | 0.044* | 0.192 (0.030-1.247) | 0.084 | - | - | 0.121 (0.021-0.691) | 0.018* | 0.139 (0.022-0.863) | 0.034* | - | - | 0.203 (0.039-1.058) | 0.058 | 0.248 (0.043-1.419) | 0.117 |

*means P < 0.05; **means P < 0.01

Abbreviations: OR, odds ratio; CI, confidence interval; EAT, early antithrombotic therapy; MT, mechanical thrombectomy; NIHSS, National Institutes of Health Stroke Scale; mRS, modified Rankin Scale; PCHD, post-thrombectomy cerebral hyperdensity; ECASS, European Cooperative Acute Stroke Study; IVT, intravenous thrombolysis.

Model 1 adjusted for age and sex. Model 2 additionally adjusted for low density on CT after MT, early brain edema, IVT before MT, dyslipidemia, NIHSS before MT, number of retrieval attempts, and PCHD type (ECASS III). Model 3 replaced NIHSS before MT with NIHSS after MT.

Complete-case (CC): patients with observed outcome. Discharge-imputed (DI): missing 90-day mRS was assigned the discharge mRS. Multiple imputation by chained equations (MICE): m=20; estimates combined by Rubin's rules. NIHSS after MT were not imputed; Model 3 therefore has fewer patients. CC: Models 1–2 n=129, Model 3 n=117. DI and MICE: Models 1–2 n=141, Model 3 n=127.

Table S4. Full multivariable logistic regression for any 24-hour hyperdensity.

| **Variable** | **Before multiple imputation (n=135)** | | | | | | **After multiple imputation (n=141)** | | | | | |
| --- | --- | --- | --- | --- | --- | --- | --- | --- | --- | --- | --- | --- |
|  | **Model 1** | | **Model 2** | | **Model 3** | | **Model 1** | | **Model 2** | | **Model 3** | |
|  | **OR (95% CI)** | **P** | **OR (95% CI)** | **P** | **OR (95% CI)** | **P** | **OR (95% CI)** | **P** | **OR (95% CI)** | **P** | **OR (95% CI)** | **P** |
| Early antithrombotic treatment | 0.462 (0.229-0.936) | 0.032* | 0.586 (0.245-1.402) | 0.230 | 0.617 (0.247-1.539) | 0.301 | 0.438 (0.218-0.880) | 0.020* | 0.582 (0.243-1.393) | 0.224 | 0.616 (0.247-1.534) | 0.298 |
| Male | 0.838 (0.383-1.834) | 0.658 | 0.995 (0.373-2.657) | 0.993 | 0.881 (0.316-2.457) | 0.809 | 0.824 (0.381-1.780) | 0.622 | 1.018 (0.382-2.716) | 0.971 | 0.896 (0.322-2.490) | 0.833 |
| Age | 1.004 (0.978-1.032) | 0.761 | 0.993 (0.956-1.032) | 0.735 | 1.005 (0.965-1.047) | 0.796 | 1.007 (0.981-1.035) | 0.601 | 0.994 (0.956-1.033) | 0.746 | 1.005 (0.965-1.047) | 0.805 |
| NIHSS score before MT | - | - | 1.037 (0.957-1.124) | 0.378 | - | - | - | - | 1.036 (0.956-1.124) | 0.386 | - | - |
| NIHSS score after MT (post-procedure) | - | - | - | - | 1.007 (0.923-1.098) | 0.877 | - | - | - | - | 1.006 (0.922-1.097) | 0.894 |
| IVT before MT | - | - | 1.117 (0.429-2.905) | 0.821 | 0.891 (0.321-2.472) | 0.824 | - | - | 1.120 (0.432-2.902) | 0.816 | 0.881 (0.318-2.440) | 0.808 |
| Dyslipidemia | - | - | 1.242 (0.501-3.080) | 0.640 | 1.565 (0.591-4.146) | 0.367 | - | - | 1.229 (0.494-3.054) | 0.657 | 1.557 (0.588-4.125) | 0.373 |
| Early brain edema | - | - | 2.229 (0.565-8.786) | 0.252 | 2.503 (0.546-11.472) | 0.237 | - | - | 2.354 (0.594-9.336) | 0.223 | 2.541 (0.551-11.716) | 0.232 |
| PCHD type (ECASS III, HI2 vs HI1) | - | - | 10.292 (3.385-31.291) | <0.001** | 10.833 (3.486-33.669) | <0.001** | - | - | 10.295 (3.373-31.424) | <0.001** | 10.748 (3.450-33.485) | <0.001** |
| Low density on CT after MT |  |  |  |  |  |  |  |  |  |  |  |  |
| Without low density on CT | - | - | Reference |  | Reference |  | - | - | Reference |  | Reference |  |
| Low density < 1/3 vascular territory | - | - | 2.892 (1.048-7.982) | 0.040* | 2.546 (0.896-7.234) | 0.080 | - | - | 2.866 (1.032-7.960) | 0.043* | 2.517 (0.883-7.178) | 0.084 |
| Low density > 1/3 vascular territory | - | - | 2.548 (0.587-11.055) | 0.212 | 2.684 (0.562-12.830) | 0.216 | - | - | 2.480 (0.574-10.710) | 0.224 | 2.685 (0.561-12.852) | 0.216 |
| Number of retrieval attempts |  |  |  |  |  |  |  |  |  |  |  |  |
| 1 | - | - | Reference |  | Reference |  | - | - | Reference |  | Reference |  |
| 2 | - | - | 0.476 (0.160-1.416) | 0.182 | 0.537 (0.176-1.635) | 0.273 | - | - | 0.469 (0.158-1.393) | 0.173 | 0.537 (0.177-1.636) | 0.274 |
| 3 | - | - | 1.119 (0.341-3.673) | 0.852 | 1.488 (0.408-5.422) | 0.547 | - | - | 1.126 (0.341-3.726) | 0.845 | 1.501 (0.407-5.531) | 0.542 |
| ≥4 | - | - | 2.173 (0.448-10.537) | 0.335 | 1.825 (0.353-9.445) | 0.473 | - | - | 2.187 (0.459-10.434) | 0.326 | 1.903 (0.371-9.757) | 0.440 |

*means P < 0.05; **means P < 0.01

Abbreviations: OR, odds ratio; CI, confidence interval; EAT, early antithrombotic therapy; MT, mechanical thrombectomy; NIHSS, National Institutes of Health Stroke Scale; mRS, modified Rankin Scale; PCHD, post-thrombectomy cerebral hyperdensity; ECASS, European Cooperative Acute Stroke Study; IVT, intravenous thrombolysis.

Model 1 adjusted for age and sex. Model 2 additionally adjusted for low density on CT after MT, early brain edema, IVT before MT, dyslipidemia, NIHSS before MT, number of retrieval attempts, and PCHD type (ECASS III). Model 3 replaced NIHSS before MT with NIHSS after MT.

Complete-case (CC): patients with observed outcome. Discharge-imputed (DI): missing 90-day mRS was assigned the discharge mRS. Multiple imputation by chained equations (MICE): m=20; estimates combined by Rubin's rules. NIHSS after MT were not imputed; Model 3 therefore has fewer patients. CC: Models 1–2 n=135 (six patients without 24-h CT excluded), Model 3 n=123. MICE: Models 1–2 n=141 (six missing 24-h hyperdensity values imputed), Model 3 n=127. No discharge-imputation analysis was performed.

Table S5. Ordinal logistic regression (proportional odds) for ΔmRS shift toward improvement.

| **Variable** | **Before imputing mRS scores (n=129)** | | | | | | **After imputing with discharge mRS scores (n=141)** | | | | | | **After multiple imputation (n=141)** | | | | | |
| --- | --- | --- | --- | --- | --- | --- | --- | --- | --- | --- | --- | --- | --- | --- | --- | --- | --- | --- |
|  | **Model 1** | | **Model 2** | | **Model 3** | | **Model 1** | | **Model 2** | | **Model 3** | | **Model 1** | | **Model 2** | | **Model 3** | |
|  | **OR (95% CI)** | **P** | **OR (95% CI)** | **P** | **OR (95% CI)** | **P** | **OR (95% CI)** | **P** | **OR (95% CI)** | **P** | **OR (95% CI)** | **P** | **OR (95% CI)** | **P** | **OR (95% CI)** | **P** | **OR (95% CI)** | **P** |
| Early antithrombotic treatment | 1.772 (0.942-3.335) | 0.076 | 2.049 (1.036-4.052) | 0.039* | 2.054 (1.014-4.159) | 0.046* | 1.882 (1.035-3.423) | 0.038* | 1.919 (1.006-3.660) | 0.048* | 1.997 (1.010-3.948) | 0.047* | 2.014 (1.098-3.696) | 0.024* | 2.239 (1.146-4.376) | 0.018* | 2.113 (1.060-4.215) | 0.034* |
| Male | 0.960 (0.478-1.928) | 0.908 | 0.973 (0.473-2.003) | 0.941 | 1.082 (0.509-2.300) | 0.838 | 0.964 (0.504-1.843) | 0.913 | 0.935 (0.475-1.841) | 0.845 | 1.138 (0.558-2.319) | 0.722 | 0.965 (0.490-1.900) | 0.918 | 0.955 (0.463-1.970) | 0.900 | 1.089 (0.513-2.313) | 0.824 |
| Age | 0.997 (0.973-1.022) | 0.813 | 0.993 (0.965-1.022) | 0.631 | 1.013 (0.983-1.044) | 0.394 | 0.995 (0.974-1.017) | 0.651 | 0.985 (0.960-1.012) | 0.278 | 1.001 (0.974-1.029) | 0.922 | 0.998 (0.975-1.021) | 0.859 | 0.989 (0.961-1.018) | 0.473 | 1.006 (0.975-1.037) | 0.721 |
| NIHSS score before MT | - | - | 1.090 (1.024-1.159) | 0.006** | - | - | - | - | 1.093 (1.031-1.158) | 0.003** | - | - | - | - | 1.100 (1.034-1.170) | 0.002** | - | - |
| NIHSS score after MT (post-procedure) | - | - | - | - | 1.006 (0.944-1.072) | 0.855 | - | - | - | - | 1.016 (0.957-1.078) | 0.611 | - | - | - | - | 1.016 (0.954-1.082) | 0.621 |
| IVT before MT | - | - | 0.926 (0.453-1.894) | 0.833 | 0.777 (0.362-1.664) | 0.516 | - | - | 0.833 (0.424-1.635) | 0.595 | 0.755 (0.370-1.539) | 0.439 | - | - | 0.872 (0.428-1.777) | 0.706 | 0.786 (0.372-1.662) | 0.529 |
| Dyslipidemia | - | - | 1.255 (0.619-2.548) | 0.529 | 1.209 (0.592-2.469) | 0.603 | - | - | 1.173 (0.593-2.318) | 0.647 | 1.210 (0.602-2.431) | 0.593 | - | - | 1.237 (0.598-2.561) | 0.567 | 1.177 (0.574-2.414) | 0.656 |
| Early brain edema | - | - | 0.182 (0.061-0.543) | 0.002** | 0.301 (0.090-1.008) | 0.052 | - | - | 0.233 (0.087-0.622) | 0.004** | 0.386 (0.134-1.111) | 0.077 | - | - | 0.229 (0.080-0.658) | 0.006** | 0.359 (0.116-1.117) | 0.077 |
| PCHD type (ECASS III, HI2 vs HI1) | - | - | 0.793 (0.360-1.749) | 0.566 | 1.045 (0.463-2.359) | 0.916 | - | - | 0.837 (0.386-1.814) | 0.652 | 1.061 (0.476-2.364) | 0.885 | - | - | 0.843 (0.384-1.849) | 0.669 | 1.033 (0.461-2.317) | 0.937 |
| Low density on CT after MT |  |  |  |  |  |  |  |  |  |  |  |  |  |  |  |  |  |  |
| Without low density on CT | - | - | Reference |  | Reference |  | - | - | Reference |  | Reference |  | - | - | Reference |  | Reference |  |
| Low density < 1/3 vascular territory | - | - | 0.249 (0.111-0.560) | <0.001** | 0.239 (0.104-0.549) | <0.001** | - | - | 0.273 (0.125-0.597) | 0.001** | 0.273 (0.121-0.612) | 0.002** | - | - | 0.249 (0.111-0.560) | <0.001** | 0.264 (0.115-0.609) | 0.002** |
| Low density > 1/3 vascular territory | - | - | 0.148 (0.045-0.479) | 0.001** | 0.148 (0.041-0.533) | 0.003** | - | - | 0.147 (0.049-0.446) | <0.001** | 0.153 (0.046-0.512) | 0.002** | - | - | 0.139 (0.044-0.444) | <0.001** | 0.153 (0.043-0.538) | 0.003** |
| Number of retrieval attempts |  |  |  |  |  |  |  |  |  |  |  |  |  |  |  |  |  |  |
| 1 | - | - | Reference |  | Reference |  | - | - | Reference |  | Reference |  | - | - | Reference |  | Reference |  |
| 2 | - | - | 0.532 (0.223-1.271) | 0.155 | 0.490 (0.201-1.195) | 0.117 | - | - | 0.481 (0.206-1.121) | 0.090 | 0.477 (0.199-1.143) | 0.097 | - | - | 0.556 (0.235-1.318) | 0.183 | 0.502 (0.207-1.217) | 0.127 |
| 3 | - | - | 0.397 (0.165-0.953) | 0.039* | 0.263 (0.105-0.656) | 0.004** | - | - | 0.385 (0.168-0.880) | 0.024* | 0.257 (0.107-0.615) | 0.002** | - | - | 0.381 (0.158-0.917) | 0.031* | 0.252 (0.100-0.632) | 0.003** |
| ≥4 | - | - | 0.317 (0.111-0.902) | 0.031* | 0.282 (0.088-0.910) | 0.034* | - | - | 0.242 (0.093-0.629) | 0.004** | 0.219 (0.077-0.621) | 0.004** | - | - | 0.282 (0.103-0.773) | 0.014* | 0.257 (0.085-0.775) | 0.016* |

*means P < 0.05; **means P < 0.01

Abbreviations: OR, odds ratio; CI, confidence interval; EAT, early antithrombotic therapy; MT, mechanical thrombectomy; NIHSS, National Institutes of Health Stroke Scale; mRS, modified Rankin Scale; PCHD, post-thrombectomy cerebral hyperdensity; ECASS, European Cooperative Acute Stroke Study; IVT, intravenous thrombolysis.

Model 1 adjusted for age and sex. Model 2 additionally adjusted for low density on CT after MT, early brain edema, IVT before MT, dyslipidemia, NIHSS before MT, number of retrieval attempts, and PCHD type (ECASS III). Model 3 replaced NIHSS before MT with NIHSS after MT.

PCHD type were not imputed. Complete-case (CC): patients with observed outcome. Discharge-imputed (DI): missing 90-day mRS was assigned the discharge mRS. Multiple imputation by chained equations (MICE): m=20; estimates combined by Rubin's rules. NIHSS after MT were not imputed; Model 3 therefore has fewer patients. CC: Models 1–2 n=129, Model 3 n=117. DI and MICE: Models 1–2 n=141, Model 3 n=127.
